# Prevalence and Determinants of Polypharmacy: A Cross‐Sectional Study Among Elderly Patients With Cardiovascular Diseases in Outpatient Clinics

**DOI:** 10.1155/sci5/6641747

**Published:** 2025-12-18

**Authors:** Negin Faramarzi Nasab, Shahab Rezaeian, Saeed Ezadi, Alireza Khatony

**Affiliations:** ^1^ Student Research Committee, Kermanshah University of Medical Sciences, Kermanshah, Iran, kums.ac.ir; ^2^ Infectious Diseases Research Centre, Health Policy and Promotion Institute, Kermanshah University of Medical Sciences, Kermanshah, Iran, kums.ac.ir; ^3^ Social Development and Health Promotion Research Center, Health Policy and Promotion Institute, Kermanshah University of Medical Sciences, Kermanshah, Iran, kums.ac.ir

**Keywords:** cardiovascular diseases, elderly, polypharmacy, prevalence

## Abstract

Polypharmacy, commonly defined as the concurrent use of five or more medications, is a pressing public health concern among older adults with cardiovascular disease, given its strong association with adverse drug–drug interactions, medication errors, and increased hospitalization rates. Despite its clinical significance, evidence from low‐ and middle‐income countries, including Iran, remains limited. This study sought to determine the prevalence of polypharmacy and identify its key determinants among elderly cardiovascular patients in Kermanshah, Iran. In this cross‐sectional survey of 426 participants, eligible participants were recruited using convenience sampling, and demographic, clinical, and medication‐related data were collected using structured questionnaires. Statistical analyses, including chi‐square tests and multivariate logistic regression, were employed to examine associations. The findings revealed that 60.1% of participants were taking five to nine medications daily. Polypharmacy was significantly associated with lower educational attainment and income, prolonged treatment duration, hospitalization history, multimorbidity, insufficient pharmacist counseling, and limited knowledge of medications and their potential adverse effects. Multivariate analysis identified the number of chronic conditions (AOR: 5.05, *p* < 0.001), hospitalization history (AOR: 5.15, *p* < 0.001), and poor medication knowledge (AOR: 6.48, *p* = 0.017), as independent predictors. These results highlight the urgent need for targeted, evidence‐based interventions, particularly pharmacist‐led counseling and patient education programs, to mitigate the burden and adverse clinical outcomes of polypharmacy, especially in resource‐constrained healthcare settings.

## 1. Introduction

The global population is aging rapidly, with projections indicating that 1.5 billion people worldwide will be aged 65 or older by 2050 [1]. This demographic shift is mirrored in Iran, where individuals aged 60 and above constituted over 10% of the total population in 2022 [2]. Among this growing elderly population, cardiovascular diseases (CVDs) are a leading cause of morbidity and mortality [3]. CVDs are particularly relevant to polypharmacy research because their management typically requires long‐term and multidrug therapeutic regimens, which place elderly patients at substantially higher risk of inappropriate medication use compared with other chronic illnesses [4, 5]. This clinical complexity makes elderly CVD patients a priority population for investigating polypharmacy.

Polypharmacy, commonly defined as the concurrent use of five or more medications, including both prescription and over‐the‐counter drugs [6], can lead to adverse health outcomes in elderly individuals [7]. These include adverse drug events such as kidney and liver damage, drug interactions, falls, reduced treatment adherence, diminished quality of life, and higher rates of hospitalization and mortality [8–12]. The reported prevalence of polypharmacy in the elderly varies widely, ranging from 10% to 90% across different studies [13–15]. This variability likely reflects differences in study populations, definitions of polypharmacy, and healthcare contexts. For example, a 2023 systematic review found a 37.1% prevalence of polypharmacy among elderly Ethiopians, while studies in Saudi Arabia (2023) and Iran (2022) reported prevalence of 44.6% and 9.5%, respectively [7, 16, 17].

Recent evidence from Iranian cohorts provides more context‐specific insights into polypharmacy among elderly CVD patients. The Pars Cohort study reported a polypharmacy prevalence of 38.9% among patients with CVDs and 7.1% among those without CVDs [18]. Similarly, the Khuzestan Comprehensive Health Study observed very low rates of polypharmacy among elderly people (1.5%) and among patients with heart diseases (4.7%) [19]. National health insurance data further showed that among individuals aged 65 years and older, 74.9% experienced cumulative polypharmacy over a 6‐month period and 64.6% over a 1‐month period and 7.6% experienced consecutive polypharmacy [20]. Furthermore, in the Persian cohort, the prevalence of polypharmacy was 9.5%, and the risk of polypharmacy in patients with four or more CVDs was 49‐fold higher than in those without CVDs [21]. These data reinforce that CVD patients constitute one of the most medication‐intensive patient groups in Iran, underscoring the importance of focusing specifically on this population.

Several factors contribute to polypharmacy, including demographic characteristics (e.g., age, gender, and socioeconomic status), health status (e.g., number of chronic conditions), and access to healthcare services [21]. While age and the presence of multiple chronic diseases are consistently identified as key risk factors, the influence of other factors, such as gender and socioeconomic status, remains less clear [13, 22, 23]. Conflicting findings have been reported regarding the association between gender and polypharmacy [13, 16, 24–27], highlighting the need for further research.

Given the potential harms associated with polypharmacy, reducing inappropriate medication use is a crucial public health priority. This aligns with the World Health Organization’s third global patient safety challenge, “Medication Without Harm” [28]. Effectively addressing polypharmacy is also a key strategy for preventing and managing frailty in older adults [29].

Considering the high medication burden inherent to CVD management and the variation reported in Iranian studies, elderly patients with CVDs represent a clinically important yet understudied group in the context of polypharmacy. This study aimed to determine the prevalence of polypharmacy and identify its associated factors among elderly patients with CVDs in Kermanshah, Western Iran. While the study focuses on a particular geographic area, the findings can contribute to the broader understanding of polypharmacy in elderly CVD patients and inform the development of targeted interventions in similar settings internationally. Specifically, this study sought to answer the following research questions: what is the prevalence of polypharmacy among elderly CVD patients in this region of Iran? and what factors are associated with polypharmacy in this population?

The findings of this study will inform strategies to optimize medication use and improve health outcomes among elderly individuals with CVDs, with potential applicability to other regions facing similar demographic and health challenges.

## 2. Materials and Methods

### 2.1. Study Design and Guidelines

This cross‐sectional analytical study was conducted following the Strengthening the Reporting of Observational Studies in Epidemiology (STROBE) guidelines [30].

### 2.2. Study Setting

The study was conducted at outpatient clinics affiliated with Kermanshah University of Medical Sciences in Western Iran. These seven clinics provide specialized cardiovascular care, including cardiologist consultations, diagnostic tests (e.g., electrocardiography, echocardiography, and exercise stress tests), and follow‐up for chronic patients. Data were collected from February 5, 2022, to March 12, 2023.

### 2.3. Study Population and Sampling

Eligible participants were elderly patients (≥ 65 years) with a confirmed diagnosis of CVD managed by a cardiologist, able to read and write, and providing informed consent, with at least one year since diagnosis. The exclusion criterion was an incomplete questionnaire. Eligible participants were recruited using convenience sampling. The sample size was calculated based on a previously reported prevalence of polypharmacy of 52.3% [31], a precision of 5%, and a 95% confidence level, resulting in a required sample size of 383. This was increased to 426 to account for a 10% anticipated nonresponse rate.

### 2.4. Variables

CVDs included all physician‐diagnosed cardiovascular conditions among clinic attendees, specifically hypertension, ischemic heart disease, heart failure, valvular heart disease, peripheral vascular disease, and cardiac arrhythmias, consistent with standard clinical classifications [32, 33]. Polypharmacy was defined as the concurrent use of five or more medications, including both prescription and over‐the‐counter drugs; herbal medications were not included in this count, based on widely accepted definitions [7]. Variables assessed included demographic characteristics (age, gender, marital status, education level, income, living situation, and lifestyle behaviors), clinical characteristics (number of chronic diseases, duration of CVD treatment, history of hospitalization, history of falls, and number of treating physicians), and medication‐related factors (list of all medications used, medication classes according to the Anatomical Therapeutic Chemical (ATC) classification system, source of medication counseling, awareness of appropriate medication use, and awareness of potential medication side effects). The prevalence of polypharmacy was calculated as the proportion of participants taking five or more medications.

### 2.5. Data Collection

Data were collected using a structured, researcher‐developed questionnaire informed by previous research [9, 22, 27, 32]. To evaluate content validity, both qualitative and quantitative content analysis methods were used. In the qualitative assessment, content validity was assessed by 12 faculty members at Kermanshah University of Medical Sciences with expertise in pharmacology, nursing, cardiology, internal medicine, and geriatrics. They reviewed the questionnaire for relevance, clarity, and comprehensiveness, and their feedback was used to refine it. In the quantitative phase, the content validity ratio and content validity index were calculated, and all items met the acceptable thresholds. To assess reliability, Cronbach’s alpha was used. The questionnaire was administered to 30 elderly patients similar to the study population, yielding an alpha coefficient of 0.80, indicating good internal consistency.

The questionnaire comprised three sections: demographic and social characteristics (seven items), health status (five items), and medication use. To assess economic status, participants were asked “how do you evaluate your income level?” with response options “sufficient to cover living expenses” or “insufficient to cover living expenses.” The health status section included questions such as “how long have you been undergoing treatment for CVD?”, “have you ever experienced a fall?”, and “how many specialists are managing your treatment?” The medication use section included a list of current medications (prescription and OTC), drug classes based on the ATC classification, sources of medication consultation, awareness of correct medication usage, and awareness of potential side effects. Sample questions included “are you familiar with the correct method of taking your medications?” and “are you aware of the potential side effects of your medications?”

Participants were approached by the researcher after their appointments with cardiologists or geriatricians and completed the questionnaire on‐site. Several measures ensured data quality: clear explanation of study objectives, simple instructions for completing the questionnaire, emphasis on confidentiality, ample time for responses, and regular verification of data entered into STATA.

### 2.6. Data Analysis

Data analysis was performed using STATA Version 14. Descriptive statistics (frequencies, percentages, means, and standard deviations) were used to summarize participant characteristics. Associations between polypharmacy and categorical variables were assessed using the chi‐square test, and continuous variables were analyzed using the independent *t*‐test. Univariate and multivariate logistic regression analyses were conducted to identify factors associated with polypharmacy. The following variables were included in the multivariate model based on their significance (*p* < 0.2) in the bivariate analyses: education level, income, treatment duration, hospitalization history, adherence to physician instructions, awareness of proper medication use, awareness of medication side effects, number of chronic conditions, and source of medication counseling. Adjusted odds ratios with 95% confidence intervals were reported, and a *p* value < 0.05 was considered statistically significant.

### 2.7. Use of Artificial Intelligence Tools

During the preparation of this manuscript, the authors used ChatGPT (OpenAI, USA) solely for improving the clarity, grammar, and readability of the text. No generative AI tools were used for study design, data collection, data analysis, interpretation of results, or generation of scientific content. All AI‐assisted text was thoroughly reviewed and edited by the authors, who take full responsibility for the integrity and accuracy of the final manuscript.

### 2.8. Ethical Considerations

The study was approved by the Ethics Committee of Kermanshah University of Medical Sciences (protocol code IR.KUMS.REC.1400.271). All participants provided written informed consent after receiving a thorough explanation of the study objectives and assurances of confidentiality. Participants were informed of their right to withdraw at any time.

## 3. Results

### 3.1. Participant Characteristics

The response rate was 100% (*n* = 426). The mean age was 71.4 ± 5.7 years, with the majority (78.6%) between 65 and 75 years. Over half of the participants were female (53.1%) and married (55.6%). Most participants resided in urban areas (74.9%). The majority (90.1%) had completed primary or secondary education, while 16.9% lived alone. A substantial proportion (69.5%) reported insufficient monthly income (Table 1).

**Table 1:** Demographics of elderly patients with cardiovascular disease by polypharmacy.

Variables		Total, *n* (%)	Polypharmacy		Test results
			Yes, *n* (%)	No, *n* (%)	
Gender	Male	200 (46.9)	120 (60.0)	80 (40.0)	*X* ^2^ = 0.11 *p* = 0.739
	Female	226 (53.1)	132 (58.4)	94 (41.6)	

Age (in years)	65–75	335 (78.6)	193 (57.6)	142 (42.4)	*X* ^2^ = 1.54 *p* = 0.214
	> 75	91 (21.4)	59 (64.8)	32 (35.2)	

Marital status	Single	17 (4.0)	9 (52.9)	8 (47.1)	*X* ^2^ = 2.94 *p* = 0.402
	Married	237 (55.6)	141 (59.5)	96 (40.5)	
	Divorced	9 (2.1)	3 (33.3)	6 (66.7)	
	Widowed	163 (38.3)	99 (60.7)	64 (39.3)	

Education	Less than high school	384 (90.1)	237 (61.7)	147 (38.3)	*X* ^2^ = 10.59 *p* = 0.001
	High school and above	42 (9.9)	15 (35.7)	27 (64.3)	

Place of residence	Urban	319 (74.9)	185 (58.0)	134 (42.0)	*X* ^2^ = 0.71 *p* = 0.400
	Rural	107 (25.1)	67 (62.6)	40 (37.4)	

Living situation	Living alone	72 (16.9)	40 (55.6)	32 (44.4)	*X* ^2^ = 0.46 *p* = 0.495
	Living with spouse and/or children	354 (83.1)	212 (59.9)	142 (40.1)	

Monthly income	Sufficient income	130 (30.5)	66 (50.8)	64 (49.2)	*X* ^2^ = 5.44 *p* = 0.020
	Insufficient income	296 (69.5)	186 (62.8)	110 (37.2)	

### 3.2. Clinical and Medication‐Related Characteristics

Participants had been receiving treatment for CVDs for an average of 8.1 ± 6.6 years. The most common CVD diagnoses were hypertension (60.1%) and ischemic heart disease (43.2%). Common comorbidities included type 2 diabetes (27.5%) and hyperlipidemia (17.6%). Most participants (67.4%) had two or three chronic conditions. A history of falls was reported by 10.1% of participants, and 75.8% had been hospitalized at least once. Nearly half (44.8%) were being managed by two specialists. Physicians were the primary source of medication counseling for 50.2% of participants. While 70.7% reported awareness of proper medication use, only 16.4% were aware of potential medication side effects (Table 2).

**Table 2:** Clinical and medication profiles of elderly patients with cardiovascular disease, stratified by polypharmacy.

Variables		Total, *n* (%)	Polypharmacy		Test results
			Yes, *n* (%)	No, *n* (%)	
Duration of treatment (in years)	> 5	203 (47.6)	140 (69.0)	63 (31.0)	*X* ^2^ = 15.44 *p* < 0.001
	≥ 5	223 (52.4)	112 (50.2)	111 (49.8)	

Number of chronic diseases	≤ 1	60 (14.1)	28 (46.7)	32 (53.3)	*X* ^2^ = 27.74 *p* < 0.001
	2‐3	287 (67.4)	157 (54.7)	130 (45.3)	
	> 3	79 (18.5)	67 (84.8)	12 (15.2)	

History of falls	Yes	43 (10.1)	31 (72.0)	12 (28.0)	*X* ^2^ = 3.31 *p* = 0.069
	No	383 (89.9)	221 (57.7)	162 (42.3)	

Hospitalization	Yes	323 (75.8)	221 (68.4)	102 (31.6)	*X* ^2^ = 47.47 *p* < 0.001
	No	103 (24.2)	31 (30.1)	72 (69.9)	

Number of specialists under supervision	1	188 (44.1)	105 (55.9)	83 (44.1)	*X* ^2^ = 1.52 *p* < 0.001
	≥ 2	238 (55.9)	147 (61.8)	91 (38.2)	

Knowledge of how to take medication	Yes	301 (70.7)	146 (48.5)	155 (51.5)	*X* ^2^ = 48.15 *p* < 0.001
	No	125 (29.3)	106 (85.0)	19 (15.0)	

Being informed about side effects	Yes	70 (16.4)	20 (28.6)	50 (71.4)	*X* ^2^ = 32.43 *p* < 0.001
	No	356 (83.6)	232 (65.2)	124 (34.8)	

Source of medication advice	Prescribing physician	214 (50.2)	87 (40.7)	127 (59.3)	*X* ^2^ = 62.22 *p* < 0.001
	Pharmacist	117 (27.5)	87 (74.4)	30 (25.6)	
	Friends and/or family	95 (22.3)	78 (82.1)	17 (17.9)	

*Comorbidities*					

Type 2 diabetes	Yes	117 (27.46)	77 (65.8)	40 (34.2)	*X* ^2^ = 2.96 *p* = 0.085
	No	309 (72.54)	175 (56.6)	134 (43.4)	

Hyperlipidemia	Yes	75 (17.6)	42 (56.0)	33 (44.0)	*X* ^2^ = 0.37 *p* = 0.540
	No	351 (82.4)	210 (59.8)	141 (40.2)	

Neurological disorders	Yes	71 (16.7)	47 (66.2)	24 (33.8)	*X* ^2^ = 1.75 *p* = 0.186
	No	355 (83.3)	205 (57.7)	150 (42.3)	

Renal/urinary tract diseases	Yes	30 (7.0)	22 (73.3)	8 (26.7)	*X* ^2^ = 2.70 *p* = 0.101
	No	396 (93.0)	230 (58.1)	166 (41.9)	

Gastrointestinal diseases	Yes	35 (8.2)	25 (71.4)	10 (28.6)	*X* ^2^ = 2.37 *p* = 0.123
	No	391 (91.8)	227 (58.1)	164 (41.9)	

Other chronic conditions	Yes	144 (33.8)	100 (69.4)	44 (30.6)	*X* ^2^ = 9.53 *p* = 0.002
	No	282 (66.2)	152 (53.9)	130 (46.1)	

### 3.3. Prevalence of Polypharmacy

**Figure 1:**
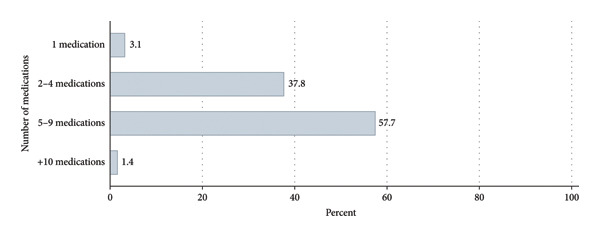
Distribution of daily medication use among elderly patients with cardiovascular diseases. Percentages of participants are shown above bars representing 1, 2–4, 5–9, and ≥ 10 medications per day. The figure shows that the majority of patients take 2–4 medications daily, while a substantial proportion (polypharmacy) takes five or more medications. Polypharmacy is defined as taking five or more medications.

**Figure 2:**
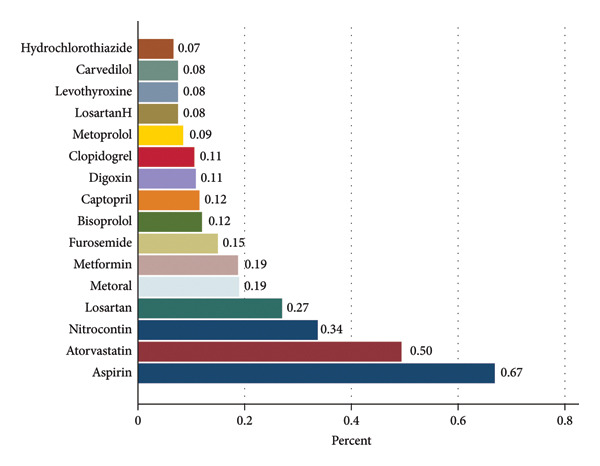
Prevalence of commonly used medications among elderly patients with cardiovascular diseases. The horizontal axis represents the percentage of participants using each medication. Aspirin (67%) and atorvastatin (50%) were the most frequently used medications, followed by nitrocontin (34%) and losartan (27%). Less commonly used medications included hydrochlorothiazide (7%), carvedilol (8%), and levothyroxine (8%). Percentages are shown at the end of each bar.

The prevalence of polypharmacy was 59.2%. The mean number of medications used per participant was 5.2 ± 2.1. Over half (57.7%) were taking five to nine medications (Figure 1). The most frequently used medications were aspirin (67.1%), atorvastatin (49.5%), nitroglycerin (33.9%), losartan (27.2%), and pantoprazole (20.2%) (Figure 2).

**Figure 3:**
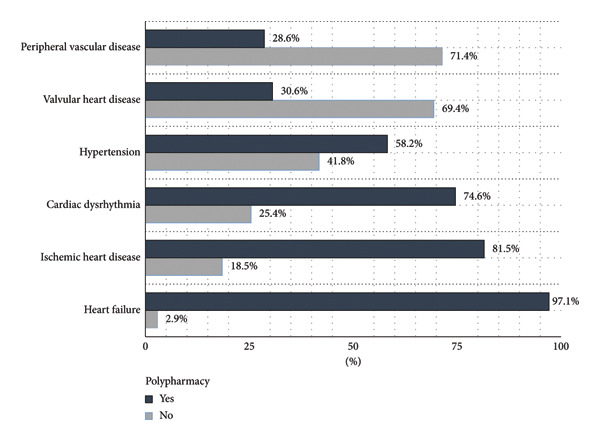
Primary diagnoses in elderly patients with cardiovascular disease according to polypharmacy.

**Figure 4:**
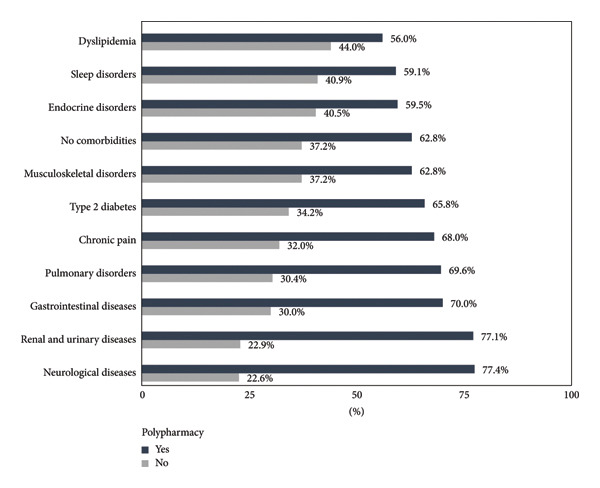
Comorbidities in elderly patients with cardiovascular disease according to polypharmacy.

Among participants with polypharmacy, the most prevalent CVD diagnoses were heart failure (97.1%), ischemic heart disease (81.5%), and cardiac dysrhythmias (74.6%) (Figure 3). The most common comorbidities in this group were neurological disorders (77.4%), renal and urinary tract diseases (77.1%), and gastrointestinal diseases (70.0%) (Figure 4).

### 3.4. Factors Associated With Polypharmacy

#### 3.4.1. Bivariate Analyses

Chi‐square tests revealed significant associations between polypharmacy and education level (*p* = 0.001) and income (*p* = 0.020). Lower education levels and insufficient income were associated with higher rates of polypharmacy (Table 1). No significant associations were found between polypharmacy and age, gender, marital status, residence, or living arrangements.

Similarly, significant associations were observed between polypharmacy and treatment duration (*p* = 0.001) and hospitalization history (*p* = 0.001). Longer treatment duration was associated with lower rates of polypharmacy, while a history of hospitalization was associated with higher rates. No significant relationships were found between polypharmacy and fall history or the number of treating physicians.

Medication knowledge and adherence: patients who reported adherence to physician instructions, awareness of proper medication use, or awareness of medication side effects had lower rates of polypharmacy (*p* = 0.001). Polypharmacy was also significantly associated with the number of chronic conditions, with higher rates observed in patients with more comorbidities (Table 2).

#### 3.4.2. Multivariate Logistic Regression Analysis

Multivariate logistic regression was performed to identify independent risk factors for polypharmacy (Table 3). The analysis showed that having more than three chronic conditions was significantly associated with a five‐fold increased risk of polypharmacy (AOR: 5.05; 95% CI: 2.01–12.69; *p* < 0.001). A history of hospitalization also significantly increased the odds of polypharmacy more than five‐fold (AOR: 5.15; 95% CI: 2.93–9.05; *p* < 0.001). Although a longer treatment duration (5 years or more) was associated with a 34% lower likelihood of polypharmacy in the bivariate analysis, this association was not statistically significant in the multivariate model (AOR: 0.66; 95% CI: 0.40–1.09; *p* = 0.110). Lack of awareness regarding proper medication use was significantly associated with a 6.5‐fold increased risk of polypharmacy (AOR: 6.48; 95% CI: 1.39–30.16; *p* = 0.017), whereas lack of awareness about medication side effects showed a trend toward increased risk, although this was not statistically significant (AOR: 1.98; 95% CI: 0.96–4.08; *p* = 0.061). Finally, receiving medication counseling from a pharmacist, compared to a physician, was significantly associated with a nearly three‐fold increased risk of polypharmacy (AOR: 2.92; 95% CI: 1.62–5.25; *p* < 0.001) (Table 3).

**Table 3:** Predictive factors for polypharmacy: a multiple regression analysis.

Variables		Crude model		Adjusted model	
		Crude OR^†^ (95% CI^ǂ^)	*p* value	AOR^¥^ (95% CI)	*p* value
Age (in years)	65–75	Ref.	—	—	—
	> 75	1.35 (0.83, 2.19)	0.215	—	—

Gender	Male	Ref.	—	—	—
	Female	0.93 (0.63, 1.37)	0.739	—	—

Marital status	Single	Ref.	—	—	—
	Married	1.03 (0.69, 1.52)	0.873	—	—

Place of residence	Urban	Ref.	—	—	—
	Rural	1.21 (0.77, 1.90)	0.400	—	—

Education	High school and above	Ref.	—	—	—
	Less than high school	2.90 (1.49, 5.63)	0.002	1.34 (0.54, 3.33)	0.518

Living situation	Living with spouse and/or children	Ref.	—	—	—
	Living alone	0.83 (0.50, 1.39)	0.496	—	—

Monthly income	Sufficient income	Ref.	—	—	—
	Insufficient income	1.63 (1.08, 2.48)	0.020	0.94 (0.52, 1.71)	0.861

Duration of treatment (in years)	5	Ref.	—	—	—
	≥ 5	0.45 (0.30, 0.67)	< 0.001	0.66 (0.40, 1.09)	0.110

Number of chronic diseases	≤ 1	Ref.	—	—	—
	2‐3	1.38 (0.79, 2.41)	0.258	1.45 (0.73, 2.87)	0.279
	> 3	6.38 (2.87, 14.15)	< 0.001	5.05 (2.01, 12.69)	< 0.001

History of falls	No	Ref.	—	—	—
	Yes	1.89 (0.94, 3.80)	0.072	1.02 (0.41, 2.55)	0.961

Hospitalization	No	Ref.	—	—	—
	Yes	5.03 (3.10, 8.14)	< 0.001	5.15 (2.93, 9.05)	< 0.001

Number of specialists under supervision	1	Ref.	—	—	—
	≥ 2	1.27 (0.86, 1.88)	0.218	—	—

Knowledge of how to take medication	Yes	Ref.	—	—	—
	No	5.92 (3.45, 10.14)	< 0.001	6.48 (1.39, 30.16)	0.017

Being informed about side effects	Yes	Ref.	—	—	—
	No	4.67 (2.66, 8.20)	< 0.001	1.98 (0.96, 4.08)	0.061

Source of medication advice	Prescribing physician	Ref.	—	—	—
	Pharmacist	4.23 (2.57, 6.95)	< 0.001	2.92 (1.62, 5.25)	< 0.001
	Friends and/or family	6.69 (3.70, 12.09)	< 0.001	0.94 (0.18, 4.84)	0.950

*R* ^2^ = 12.1%					

^†^Odds ratio.

^ǂ^Confidence interval.

^¥^Adjusted odds ratio.

## 4. Discussion

This study examined the prevalence of polypharmacy and its associated factors among elderly patients with CVDs. The observed prevalence of 59.2% is consistent with some previous studies but differs from others, reflecting variations in study populations, healthcare accessibility, study timelines, and operational definitions of polypharmacy. Within Iran, Zare et al., using data from the Pars Cohort, reported prevalence rates of 23.7% among patients with hypertension and 4.7% among those without hypertension [33]. Similarly, the KCHS reported a polypharmacy prevalence of 4.7% among patients with CVDs [19], while the AZAR Cohort documented a rate of 31.9% among individuals with CVDs [16]. These Iranian cohorts provide comparable population characteristics and similar healthcare settings to the present study, highlighting the considerable burden of polypharmacy among elderly patients with CVDs in Iran.

In neighboring countries, studies conducted in Turkey and Saudi Arabia have shown polypharmacy prevalence rates of 33% and 55% among older adults, respectively [34, 35], indicating a regional trend of high polypharmacy rates among elderly individuals with chronic conditions. Comparisons with other countries offer further insight. For example, studies from Saudi Arabia by AlAbdulKader et al. and Aljawadi et al. reported prevalence rates of 44.6% and 51.5% among elderly populations [7, 9]. Moreover, systematic reviews from Ethiopia (37.1%) [17], China (48% among older adults and 23% among outpatients) [36], and Kuwait (58.4%) [25] demonstrate both similarities and cross‐national variations, likely attributable to differences in study designs, healthcare infrastructure, population characteristics, and definitions of polypharmacy used across studies.

In our study, the most frequently used medications among elderly patients with CVDs—including aspirin, atorvastatin, nitroglycerin, losartan, and pantoprazole—were identified. Evidence from Iranian cohorts further contextualizes these findings. In the Pars Cohort, drugs affecting the renin–angiotensin system were most frequently prescribed, followed by β‐blockers [18]. Similarly, in the KCHS, patients experiencing polypharmacy most commonly used cardiovascular drugs, gastrointestinal and metabolic agents, and medications affecting the nervous system [19]. National health insurance claims data indicated that systemic corticosteroids, HMG‐CoA reductase inhibitors, and antiplatelet agents were among the most frequently prescribed drug classes [20]. In the AZAR Cohort, cardiovascular, nervous system, and endocrine medications were the most commonly used [16]. Comparisons with international studies reveal notable differences. For example, higher diuretic use in Ethiopia [27] and greater utilization of antihypertensive and antidiabetic agents in Kuwait [25] likely reflect variations in patient clinical profiles, prescribing habits, healthcare system organization, and guideline adherence. These findings suggest that the types of medications elderly patients take are shaped not only by their health conditions but also by local prescribing practices and healthcare system characteristics, emphasizing the importance of context‐specific approaches in managing polypharmacy.

Lower education was significantly associated with higher polypharmacy prevalence in our study. Evidence from Iranian cohorts further supports this association. In the Pars Cohort, polypharmacy prevalence was higher among individuals with no formal education compared to those who were literate, although this difference was not statistically significant [18]. In contrast, the AZAR Cohort reported a statistically significant association, with polypharmacy prevalence nearly twice as high among participants with no formal education compared to those with academic degree [16]. When compared internationally, these findings align with results from Devkota in Russia [37] and Assari et al. [38] and are further supported by Dadashihaji et al. among Iranian older adults [39]. These results suggest that older adults with lower education may face greater challenges in managing multiple medications and making informed treatment decisions, which can contribute to higher polypharmacy. This emphasizes the need for clear communication and tailored support for patients with lower health literacy.

In our study, individuals with insufficient income were more likely to experience higher rates of polypharmacy. Findings from Iranian cohorts further highlight the complexity of this relationship. In the Pars Cohort, individuals with higher socioeconomic status had the highest prevalence of polypharmacy, whereas those with lower socioeconomic status had the lowest rates [18]. In contrast, the KCHS found no statistically significant association between socioeconomic status and polypharmacy prevalence [19]. Comparisons with international contexts reveal inconsistent patterns. For example, Aljawadi et al. in Saudi Arabia reported that higher income was associated with increased polypharmacy [9], while Silva et al. in Brazil found that lower income combined with lack of health insurance reduced the likelihood of polypharmacy [40]. These differences underscore the complex interplay among socioeconomic conditions, healthcare access, and medication use, which can vary across healthcare systems and sociocultural environments [41]. These findings suggest that income affects medication use differently depending on context. More importantly, people’s real‐life access to healthcare—not just financial resources—shapes how many medications they take, highlighting the need for tailored support and patient‐centered interventions in polypharmacy management.

Hospitalization history was strongly associated with polypharmacy, with affected individuals exhibiting a five‐fold higher likelihood of experiencing polypharmacy. This finding is consistent with prior research indicating that hospitalization is both a contributor to and a consequence of polypharmacy [13, 14]. Hospitalization often reflects more severe or complex disease states, necessitating multiple medications. Moreover, polypharmacy increases the risk of medication‐related complications and subsequent readmissions [42, 43], creating a bidirectional cycle. These findings highlight the importance of structured post‐discharge medication review programs for elderly patients.

In our study, multimorbidity was strongly associated with polypharmacy, with individuals having more than three chronic conditions exhibiting five times the odds of polypharmacy. Evidence from Iranian cohorts further supports this relationship. In the AZAR Cohort, the prevalence of polypharmacy among individuals with more than four chronic diseases was approximately 49 times higher than among those without any chronic conditions, a difference that was statistically significant [16]. Internationally, similar patterns have been observed. Nigussie and Bayou in Ethiopia and Devkota in Russia both documented strong and significant associations between multimorbidity and polypharmacy [22, 37]. Multimorbidity inherently increases the need for multiple therapeutic regimens, thereby making polypharmacy more likely. These findings indicate that older adults with multiple chronic conditions are at particularly high risk for polypharmacy, highlighting the need for prioritized medication review, optimization strategies, and deprescribing efforts to improve patient safety and treatment outcomes.

Elderly individuals who relied on pharmacists for medication counseling were found to have nearly three times the risk of polypharmacy compared to those who consulted physicians. While Krause et al. showed that physicians are the primary source of medication information in Germany [44] and Mortazavi et al. emphasized the central role of physicians in reducing inappropriate polypharmacy in Iran [45], numerous studies highlight the effectiveness of pharmacist‐led interventions in improving medication safety [46–49]. Importantly, this finding should not be interpreted causally. It is more plausible that individuals seeking pharmacist counseling already have complex medication regimens requiring additional guidance. In this sense, reliance on pharmacists likely reflects underlying complexity rather than contributing directly to polypharmacy. Collaborative care models—such as those described by Wang et al. in Taiwan [49], involving physician prescribing and pharmacist‐led medication optimization—offer promising strategies for managing polypharmacy.

Finally, lack of awareness regarding proper medication use was associated with a markedly increased risk of polypharmacy (6.5‐fold). While some studies suggest adequate medication knowledge among older adults [44], others document substantial gaps, particularly during transitions of care such as hospital discharge [50]. Polypharmacy itself, compounded by age‐related cognitive decline, can further reduce medication understanding [51, 52]. These findings underscore the critical importance of providing clear, accessible, and consistent medication information and strengthening patient education efforts to ensure comprehension and safe medication use.

## Conflicts of Interest

The authors declare no conflicts of interest.

## Funding

This article was funded by the Kermanshah University of Medical Sciences, 10.13039/501100005317, 50000227.

## Data Availability

The data that support the findings of this study are available on request from the corresponding author. The data are not publicly available due to privacy or ethical restrictions.
